# Sexual Dimorphism of Staminate- and Pistillate-Phase Flowers of *Saponaria officinalis* (Bouncing Bet) Affects Pollinator Behavior and Seed Set

**DOI:** 10.1371/journal.pone.0093615

**Published:** 2014-04-01

**Authors:** Sandra L. Davis, Dana A. Dudle, Jenna R. Nawrocki, Leah M. Freestone, Peter Konieczny, Michael B. Tobin, Michael M. Britton

**Affiliations:** 1 University of Indianapolis, Department of Biology, Indianapolis, Indiana, United States of America; 2 DePauw University, Department of Biology, Greencastle, Indiana, United States of America; Central China Normal University, China

## Abstract

The sequential separation of male and female function in flowers of dichogamous species allows for the evolution of differing morphologies that maximize fitness through seed siring and seed set. We examined staminate- and pistillate-phase flowers of protandrous *Saponaria officinalis* for dimorphism in floral traits and their effects on pollinator attraction and seed set. Pistillate-phase flowers have larger petals, greater mass, and are pinker in color, but due to a shape change, pistillate-phase flowers have smaller corolla diameters than staminate-phase flowers. There was no difference in nectar volume or sugar content one day after anthesis, and minimal evidence for UV nectar guide patterns in staminate- and pistillate-phase flowers. When presented with choice arrays, pollinators discriminated against pistillate-phase flowers based on their pink color. Finally, in an experimental garden, in 2012 there was a negative correlation between seed set of an open-pollinated, emasculated flower and pinkness (as measured by reflectance spectrometry) of a pistillate-phase flower on the same plant in plots covered with shade cloth. In 2013, clones of genotypes chosen from the 2012 plants that produced pinker flowers had lower seed set than those from genotypes with paler flowers. Lower seed set of pink genotypes was found in open-pollinated and hand-pollinated flowers, indicating the lower seed set might be due to other differences between pink and pale genotypes in addition to pollinator discrimination against pink flowers. In conclusion, staminate- and pistillate-phase flowers of *S. officinalis* are dimorphic in shape and color. Pollinators discriminate among flowers based on these differences, and individuals whose pistillate-phase flowers are most different in color from their staminate-phase flowers make fewer seeds. We suggest morphological studies of the two sex phases in dichogamous, hermaphroditic species can contribute to understanding the evolution of sexual dimorphism in plants without the confounding effects of genetic differences between separate male and female individuals.

## Introduction

Dichogamy, the separated presentation of male and female sexual functions during flower development, is found in more than 4200 species of angiosperms [1]. The evolution of dichogamy may result from selection for the avoidance of inbreeding and/or selection for reduction of interference between the male and female functions of the flower [2]. Complex selection pressures from a variety of intrinsic and extrinsic forces such as the presence of self-incompatibility mechanisms, inflorescence structure and size, pollination mechanisms and pollinator availability, and population dynamics influence the timing and extent of dichogamy in myriad angiosperm systems (e.g, [1], [3]–[7]).

Whatever the force drives the initial evolution of dichogamy, the separation of the sexual functions within hermaphroditic flowers may allow for the evolution of differences in floral traits in the staminate- (male) and pistillate- (female) phase flowers. Differences between staminate- and pistillate-phase flowers in morphological traits are a form of sexual dimorphism. Most studies of sexual dimorphism in plants have focused on dioecious or monoecious species that produce unisexual flowers [8]–[10]. Sexual dimorphism in plants can result from sexual selection due to competition for mate acquisition via pollinator attraction and may lead to differences in secondary sexual characteristics such as petal size and color [11]–[13]. Alternatively, sexual dimorphism in dioecious species can be the result of sex-specific or viability selection between sexes [14]. Theory predicts that selection should also act differentially on male and female functions of hermaphroditic flowers [12], [15], [16]. Dichogamy may allow populations to respond to this differential selection leading to dimorphism of secondary sexual characteristics between staminate and pistillate phases of hermaphroditic flowers.

Theory also predicts that sexual selection in plants should be stronger via male function (pollen dispersal) than via female function (pollen receipt) because male mating success is more likely to be limited by the amount of pollen dispersed by pollinators, whereas female fitness may be maximized by just a few pollinator visits that bring adequate pollen amounts for full seed set [15], [17]. Hence, the evolution of attractive traits of flowers is assumed to occur primarily through selection for male fitness. Empirical studies of selection on male and female fitness in plants with hermaphroditic flowers have shown that this assumption is not always correct. In particular, when seed set is pollen limited, selection acts on floral traits through female function as well [18]–[22]. If increased pollinator visitation increases both male and female fitness, selection may result in a common phenotype for both pistillate and staminate phase flowers. Conversely, if selection is conflicting between the genders, divergent floral morphologies may arise [20].

In dichogamous species, the staminate and pistillate stages occur within the same flower, reducing the opportunity for gender-related specialization due to genetic constraints relative to species with unisexual flowers. Indeed, in some species, pollinators may select for *similarity* between the gender phases, such as when dichogamous species provide pollen as a reward for animal pollinators [1] and pistillate flowers mimic staminate-phase flowers with a false reward [20], [23]. However, in some species, dimorphism of floral morphology could result from differences in timing of gene expression or ontogenetic changes. For example, several studies have found differences in nectar production between gender phases of dichogamous species [24]–[27], generally showing that staminate-phase flowers produce more nectar than pistillate-phase flowers, as sexual selection theory would predict.

In addition to developmental changes in sex expression and nectar production, many species experience floral color change as flowers age, or due to environmental triggers such as pollination or light. Floral color change has been described in a wide variety of species (reviewed in Weiss [28]) and often serves as a mechanism for plants to retain flowers beyond their period of sexual viability while directing pollinators to flowers with rewards [29]–[30]. In a recent study by Jabbari et al [31], floral color change was shown to be associated with dichogamy in *Saponaria officinalis*. Flowers of *S. officinalis* are protandrous, and transition from a staminate phase to a pistillate phase. As flowers change gender, they also accumulate anthocyanin in their petals, and transition from white to pink. This color change is more intense when plants are exposed to sunlight [31]. Because the pistillate-phase flowers are still receptive to pollen receipt, this color change is not associated with a change to sexual inviability, as in other species that show color change. Because floral color has been shown to be a powerful cue to attract pollinators in many species [32]–[34], color change in *S. officinalis* could impact pollinator behavior towards pistillate and staminate phase flowers, which in turn could affect fitness through either male or female function.

Here we describe sexual dimorphism in several floral traits including flower size, color, and nectar production of staminate- and pistillate-phase flowers of *S. officinalis* growing in two environmental conditions: sun and shade. We then consider how diurnal pollinators respond to arrays of staminate- and pistillate-phase floral arrays. Finally, we examine how female fitness, as measured by seed set, is associated with flower color in sun and shade environments.

## Materials and Methods

### Ethics Statement

No permits were required for the described study. The plant that is the subject of this study, *Saponaria officinalis*, is not an endangered or protected species and is a weed of disturbed areas, so sampling is not restricted. Plants for this study were collected from public areas and required no permission or from the DePauw University Nature Park by permission of DePauw University (www.depauw.edu). GPS co-ordinates for the sites for this study are: Whitewater canal: 39°84′N 86°18′W, West Street: 39°74′N 86°17′W, Greenway: 39°78′N 86°19′W, People's Pathway: 39°66′N 86°79′W, and DePauw University Nature Park: 39°64′N 86°88′W.

### Study Populations

*Saponaria officinalis* is an herbaceous weed introduced to the United States from Europe that grows in disturbed areas such as along roadsides, edges of wooded areas, ditches, and stream banks. It spreads clonally by underground rhizomes with shallow root systems and produces dense clusters of inflorescences consisting of racemes that are 0.3–0.6 m in height. Plants from five different naturalized populations were used in this study: three located in Marion County, IN (Whitewater canal, West Street, and, Greenway) and two located in Putnam County, IN (People's Pathway and DePauw University Nature Park). GPS co-ordinates for each site are listed above.

### Establishment of Experimental Gardens

In the summer of 2012, an experimental garden was established at DePauw University's Nature Park and Field Laboratory in Greencastle, IN (Putnam County). Five plants (genets, hereafter called “genotypes”) were collected from each of the five different naturalized populations of *S. officinalis* described above. Each of the 25 plants was then split by cutting the underground rhizome into eight separate plants (ramets, hereafter called “clones”). Therefore, there were eight replicates of each of 25 genotypes (five genotypes from each of the five populations, eight clones of each genotype, totaling 200 plants). Eight test plots that were 2.5×2.5 m^2^ were constructed at the test site. Twenty-five plants (one clone of each genotype) were planted in each plot in a 5×5 grid pattern with 0.5 m separating each plant. Wooden frames were constructed over each plot to create either a sunny or shaded environment in an alternating, split plot design: four of the plots were covered with 60% shade cloth and the other four were covered with a transparent mesh. The cloth was draped over the top of the wooden frame with approximately 1 m left open at the bottom to allow pollinators to enter. Due to extreme drought in 2012, there were not sufficient numbers of clones of each genotype that flowered to analyze genotypic effects; therefore, analyses described below were conducted with a subset of these plants. For each genotype, one clone for which there was a complete data set was randomly selected from both the shade plots the sun plots. One genotype lacked any clones meeting this requirement, so the final data set included 48 total plants, two clones from each of 24 genotypes, one grown in a shade plot, one grown in a sun plot.

In the summer of 2013, as part of a separate experiment to examine the variation among *S. officinalis* genotypes in floral color, a second experimental garden was established using clones of genotypes that showed variation in these traits in the summer of 2012. Two sets of genotypes were chosen from the 2012 plants: four genotypes representing those whose flowers showed the least response to sun exposure (the “pale” set) and four genotypes representing those whose flowers had the greatest response to sun exposure (the “pink” set). Thirty clones of each genotype were made by splitting of the underground rhizomes. The clones were then planted into ten plots in the new experimental garden, so each genotype is represented in each plot with three replicate clones (10 plots ×3 clones/genotype/plot ×8 genotypes  = 240 plants). Five of the plots were covered by a 60% shade cloth and five plots were covered by a clear mesh fabric (sun treatment).

### Flower Size, Shape, and Color

#### Naturalized Population

In the summer of 2012, one staminate and one pistillate phase flower were collected from 30 different plants growing in the People's Pathway population. For each flower, we measured the length and width of one petal using digital calipers. Corolla diameter was determined as the maximum transverse distance across the petal display. The fresh mass of all 5 petals was taken to the nearest 0.0001 g. Next, the petals were dried in a drying oven at 60°C for a minimum of 24 hrs and weighed again to determine dry mass. Finally, anthocyanins were extracted from the dried petals following the methods of Gould et al. [35]. The amount of light absorbed by the anthocyanin extract was quantified by visible spectroscopy using an Ocean Optics Vis™ spectrometer and LoggerPro™ software. The percent absorbance was converted to absorbance at the spectral peak, relative to dry mass and solvent volume (ABSλ527nm/g/ml). Differences in flower measurements, mass, and anthocyanin concentration between staminate and pistillate phase flowers were analyzed using paired t-tests.

#### Correlation between Reflectance and Anthocyanin Concentration

**Figure 1:**
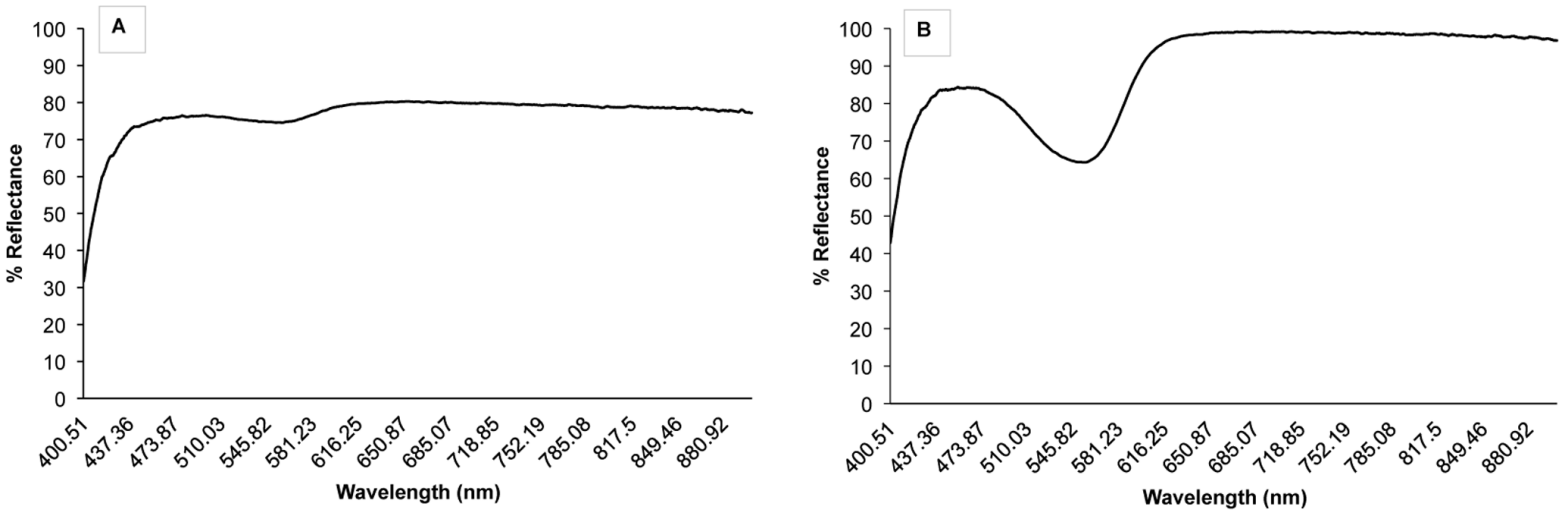
Reflectance spectra from a white staminate-phase flower, Pinkness Index (PI) = 0.0764 (A) and a pink pistillate-phase flower, PI = 0.4896 (B) of *Saponaria officinalis*.



We examined the relationship between anthocyanin concentration found in petals and the “pinkness” of the flowers as determined with an Ocean Optics Reflectance Spectrometer™ with a UV-VIS light source (200–800 nm) and fiber optic reflectance probe. We characterized floral pinkness from the reflectance spectra by calculating a “Pinkness Index” (Fig. 1). The pinkness index 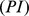 is calculated from the reflectance spectrum's two peaks (mean percent reflectance between 455–480 and 650–700 nm) and its valley (mean percent reflectance between 540–575 nm) using the following equation:

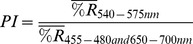



Spectra from pink flowers a have lower reflectance in the valley range, and therefore a higher pinkness index (Fig. 1). The higher the 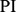 value, the pinker the flower on a scale from 0 (white) to 1 (pink).

To determine whether floral pinkness is correlated with anthocyanin concentration, and to establish whether floral pinkness differs between gender phases, we collected one staminate and one pistillate flower from 20 different plants growing in the DePauw University Nature Park. Floral pinkness for each flower was measured first. One spectrum was collected from each flower by placing the reflectance probe the center of one intact petal from each flower.

After the reflectance spectrum was collected, the fresh mass of the four remaining petals was determined and the anthocyanins were extracted and quantified as described above. The correlation between a flower's pinkness index and anthocyanin concentration was calculated as the Pearson Correlation Coefficient. Differences in pinkness index and anthocyanin concentration between staminate and pistillate flowers on a plant were evaluated with paired t-tests.

#### Floral Morphology in the Experimental Garden

To determine the effect of sun exposure on floral morphology, we collected one staminate phase and one pistillate phase flower from each clone growing in the experimental shade and sun plots of the 2012 experimental garden. Petal length, petal width, corolla diameter, and corolla mass were measured as described above. Floral pinkness and anthocyanin concentration were subsequently determined for each flower, using the methods above.

Differences between staminate and pistillate phase flowers were analyzed using a two-way ANOVA, with floral gender phase (staminate and pistillate) and treatment (shade and sun) as independent fixed effects.

#### UV Nectar Guides

The presence or absence of UV nectar guides was determined on staminate- and pistillate-phase flowers, using both a qualitative and quantitative method. To ascertain whether nectar guides were visually apparent, four flowers of different ages representing the early staminate to late pistillate stages were collected from plants growing in the DePauw University Nature Park's naturalized population. These flowers were photographed with a Nikon D90 digital SLR camera with a 24–200 mm Nikkor Lens under various combinations of light, lens, and filter conditions: natural daylight, light from a UV lamp (762 UV Ultraviolet Light Field and Lab Lamp from SIRCHIE Finger Print Labs Inc., 4-watt Longwave UV-A black light, peak wavelength at 365 nm) outside or in a darkroom, Nikon glass lens or pinhole camera lens, with or without Kodak 18A UV filter (to remove visible wavelengths).

To investigate the presence or absence of nectar guides using a quantitative method, pairs of staminate and pistillate pairs of *S. officinalis* flowers were collected from twenty-five plants in a natural population in an open field near DePauw Nature Park (in full sun). The reflectance spectra from one petal from each flower was examined at two points (distal and proximal to the flower center) using the same Ocean Optics Reflectance Spectrometer™ with a UV-VIS light source (200–800 nm) and fiberoptic reflectance probe. The UV spectra were characterized by calculating the ratio of mean reflectance values across the UV wavelengths (200–350 nm) divided by a baseline. We determined the baseline to be the average of the two peaks in the visible range (455–480 and 650–700 nm). Since the spectrophotometer collected both UV and visual wavelengths simultaneously for each flower, the visual range baseline can be used to correct any error in UV mean calculation due to calibration differences or overall reflectance differences between trials/data sets. The data were analyzed using a two-way ANOVA with the UV ratio as the dependent variable and the independent variables being floral sex and petal position.

### Nectar production and floral age

Ten individual plants representing different genotypes were transplanted from the People's Pathway population in May 2013 into 8-inch pots filled with a mixture of Metromix—™ and compost. The plants grew in the greenhouse until they began to bolt in late June. As each plant began to bolt, it was moved to a Percival growth chamber set at 25°C for 16 h (day) and 17°C for 8 hr (night).

Seven flower buds were selected on each plant, marked with jewelry tags upon opening, and monitored daily. One flower from each plant was collected to represent 1–7 days post-anthesis. Nectar from each flower was collected from the base of the flower by peeling back the calyx tube and using 10μl capillary tubes to extract all the visible nectar. Nectar volume was calculated by measuring the height of the nectar within the capillary tube (diameter  = 0.5 mm) with digital calipers. Sucrose concentration of the nectar was determined by transferring the nectar from the capillary tube to the center plate of a hand-held refractometer. Changes in nectar volume and sucrose concentration from day 1–7 were analyzed with repeated-measures ANOVA.

### Pollinator Observations

Because a previous study of *S. officinalis* demonstrated that flowers exposed to sun accumulated more anthocyanins in the pistillate stage than those kept in shade [31], we were able to manipulate the color of pistillate flowers and decouple this variable from the change in flower shape that also occurs as flowers transition from staminate to pistillate. We were then able to test pollinator responses to both flower gender and flower color. Inflorescences were collected from plants in the experimental garden and/or from surrounding populations that had either been exposed to sun or covered with shade cloth. Individual plants in naturalized populations were covered with tomato cages draped with 60% shade cloth for this experiment. Cut inflorescences in green florist tubes filled with water were arranged in test tube racks, and individual flowers were removed so that each array contained the same number of a single type of flower: either staminate white flowers, pale pistillate flowers or pink pistillate flowers.

For pollinator observations, two arrays of different flower types were placed side by side approximately 0.5 m apart. Pollinator visits were observed and recorded for twenty-minute intervals between 12:00 and 15:00 on sunny, clear days in June and July at the DePauw Nature Park adjacent to the experimental garden.

In 2012, 34 trials comparing white staminate and pink pistillate flower arrays, and 22 trials comparing white staminate arrays and pale pistillate arrays were conducted. In 2013, 27 replicates of three trial types were conducted: white staminate vs. pink pistillate arrays, white staminate vs. pale pistillate arrays, and pale pistillate vs. pink pistillate arrays. During each trial, the type of insect, which array it visited first, and how many flowers it visited in each array was recorded. An insect that visited an array was considered to be a potential pollinator if it landed on a flower and probed the interior of the flower.

The raw data were first transformed to correct for zeros by adding 0.5 and taking the square root of each data point (so it would meet the assumptions of the Student's t-test). For each trial type we used paired t-tests to compare the number of pollinator visits to each array and the number of pollinator visits per flower in each array. The ratio between initial visits to the two arrays within each trial type was tested against a null hypothesis of 50∶50 using a binomial test.

### Seed Set

#### Experimental Garden 2012

We examined seed set of hand-pollinated and open-pollinated flowers on sun- and shade-grown plants. Concurrent to the investigation of the size and color of staminate- and pistillate-phase flowers, three buds on each clone grown in the experimental garden were tagged using small jewelry tags prior to opening. The first flower was emasculated in bud and then left to be open-pollinated. The second flower was emasculated in bud and when its stigmas were exerted from the floral tube (approximately 2–3 days post-anthesis) it was hand pollinated by brushing the stamens from polliniferous flowers collected from different plants across the stigmatic surface. The third flower was left intact, and when it reached the pistillate stage was also hand pollinated using the same procedure as described for the second flower. Each flower was then allowed to mature, and the ripened fruit was collected 4–5 weeks later prior to capsule dehiscence. The number of seeds in each fruit was then counted.

Pollen limitation was estimated by comparing the number of seeds per fruit in the emasculated open-pollinated and the emasculated hand-pollinated flowers separately in the shaded plants and the sun exposed plants by a paired t-test. To determine if emasculation had a negative effect on seed production, the number of seeds per fruit in the emasculated hand-pollinated and the intact hand-pollinated flowers were compared in the shaded plants and the sun exposed plants separately by a paired t-test.

To determine the effect of floral size on seed production, the Pearson Correlation Coefficient was calculated between petal length, petal width, and corolla diameter of the pistillate-phase flower collected for color analysis and the number of seeds produced by the emasculated, open-pollinated flower described above. To determine the effects of floral color on seed production, we determined correlations between floral pinkness and anthocyanin concentration of the pistillate-phase flower collected for color analysis, and number of seeds produced by the emasculated, open-pollinated flower. Each analysis was performed separately for the shaded plants and the sun-exposed plants.

#### Experimental Garden 2013

In 2013, one staminate and one pistillate flower was collected from each clone and analyzed for color by determining the anthocyanin concentration of the petals and by calculating the pinkness index from the reflectance spectra as described above. In addition, just as in 2012, three buds on each clone were tagged and subjected to the same pollination treatments as above.

Pollen limitation was again tested for by comparing the number of seeds per fruit in the emasculated open pollinated and the emasculated hand-pollinated flowers separately in the shaded plants and the sun exposed plants by a paired t-test.

To determine the effect of floral color on seed production through pollinator visitation, a nested ANOVA was performed using the General Linear Model analysis of SPSS™. The dependent variable was number of seeds produced in the emasculated open-pollinated flower. The independent variables were treatment (sun versus shade), and genotype (1–8) nested within color set (pale versus pink). This analysis was the repeated for the emasculated hand-pollinated flowers to determine if differences in seed set among genotypes were persistent if pollen limitation was removed.

## Results

### Flower Size, Shape, and Color

**Figure 2:**
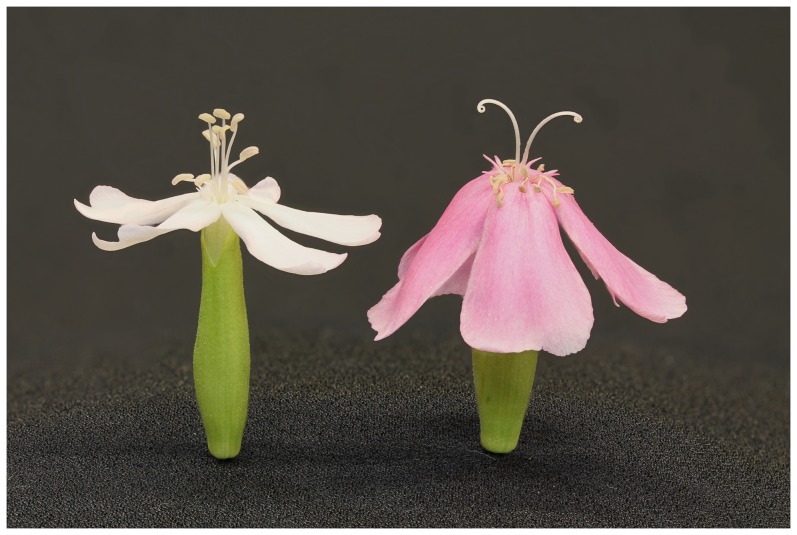
Staminate-phase (left) and pistillate-phase (right) flowers of *Saponaria officinalis*.

Individual petals of *S. officinalis* flowers grow larger as the flower transitions from the staminate phase to the pistillate phase. Measurements from flowers from plants growing in a natural population showed that petals were wider and longer in pistillate phase flowers compared to staminate flowers. Furthermore, this increase was not just due to an increase in water accumulation; pistillate flowers had higher fresh mass and higher dry mass than staminate flowers (Table 1). Despite the increase in petal size, staminate flowers had a larger display in terms of corolla diameter (Table 1). This can be explained by the fact that as the flowers transition from staminate to pistillate, the petals also reflex downward, changing the shape of the flower (Fig. 2). Finally, pistillate flowers had significantly higher anthocyanin concentrations per gram of dry mass (Table 1, Fig. 2).

**Table 1:** Differences in floral traits (mean ± s.e.) of staminate- and pistillate-phase flowers from 30 individuals of *Saponaria officinalis*.

	Staminate	Pistillate	t (df)	P
**Petal Width (mm)**	6.96±0.16	7.71±0.15	9.06 (28)	<0.001
**Petal Length (mm)**	14.30±0.17	15.02±0.19	5.29 (28)	<0.001
**Corolla Diameter (mm)**	27.27±0.42	25.06±0.57	−4.01 (28)	<0.001
**Fresh Mass (g)**	0.036±0.001	0.041±0.001	7.57 (29)	<0.001
**Dry Mass (g)**	0.005±0.0002	0.006±0.0002	2.55 (29)	0.016
**ABS_λ527nm_/g/mL**	6.74±0.76	40.31±3.74	10.03 (29)	<0.001

#### Correlation between Reflectance and Anthocyanin Concentration

**Figure 3:**
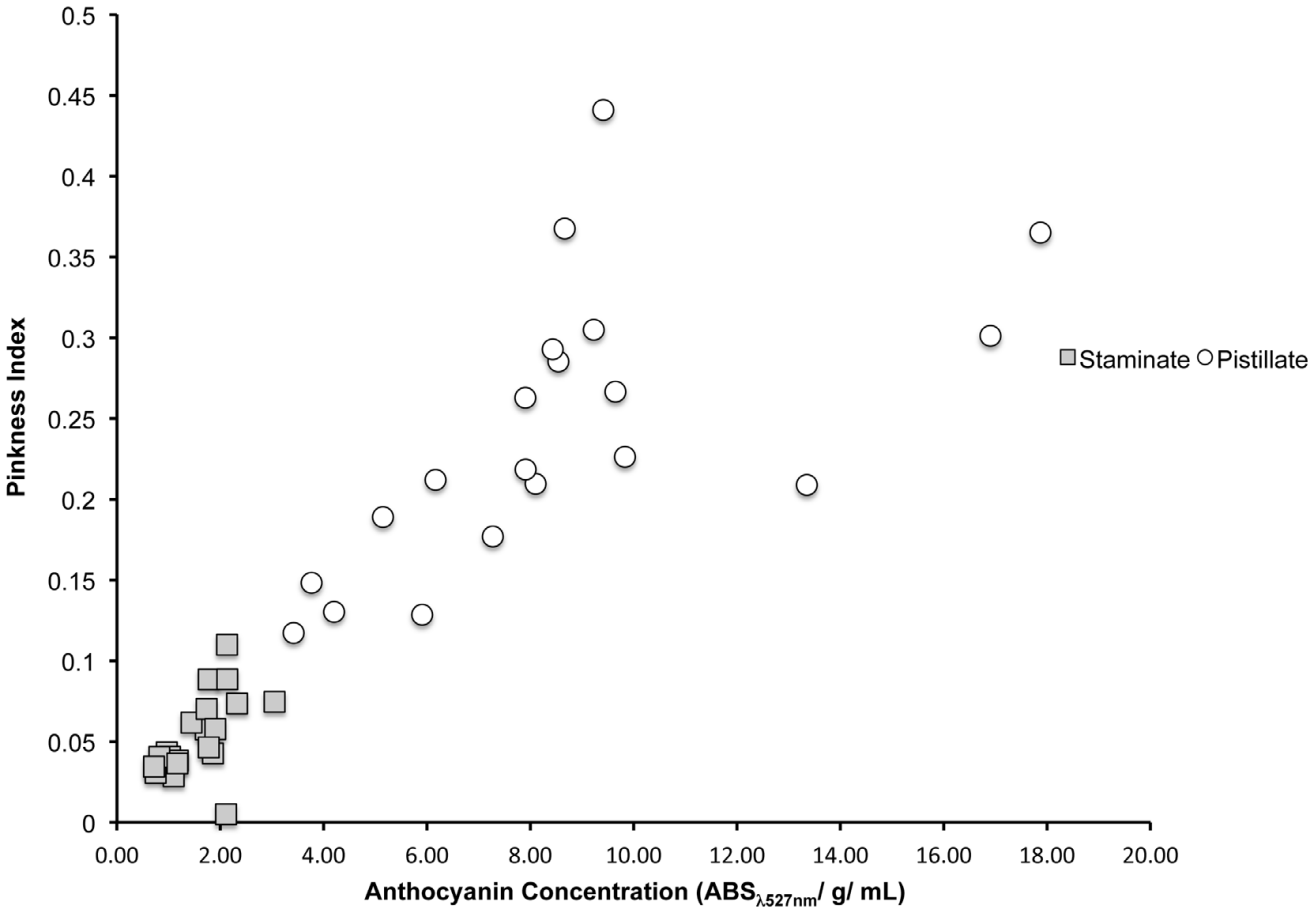
Correlation between anthocyanin concentration and pinkness index. The relationship between anthocyanin concentration and color of petals calculated as a pinkness index in staminate- and pistillate-phase flowers from 20 *S. officinalis* plants in a natural population.

Anthocyanin concentration (ABS**_λ_**_527nm_/g/mL) and the pinkness index calculated from light reflectance spectra were significantly correlated for both staminate flowers (r = 0.550, P = 0.012) and pistillate flowers (r = 0.621, P = 0.003). Staminate flowers and pistillate flowers are also significantly different for both anthocyanin concentration (t = −8.057, P<0.001) and pinkness index (t = −8.851, P<0.001; Fig. 3).

#### Floral Morphology in the Experimental Garden

**Figure 4:**
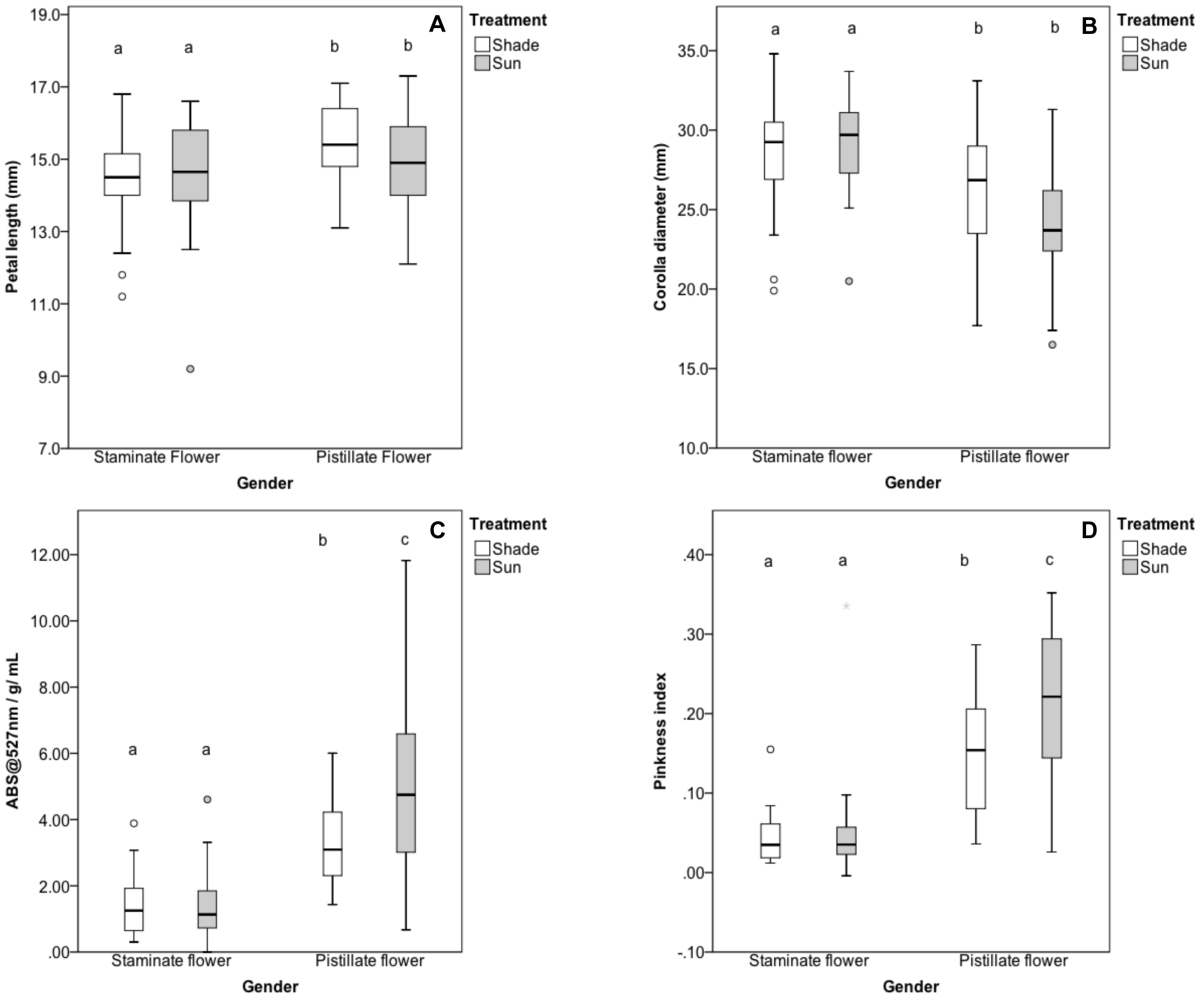
Size and color of staminate- vs. pistillate-phase flowers. Flower size as measured by petal length and corolla diameter (A and B, respectively) and flower color as measured by anthocyanin concentration and pinkness index (C and D, respectively) in staminate- and pistillate-phase flowers of *S. officinalis* growing in shaded or sun-exposed plots in an experimental garden in 2012. Boxes represent the median and quartile ranges for each variable. Open circles represent values that lie between 1.5 and 3 box lengths from the end of the box. Different letters above each box indicate significant differences (α = 0.05).

For plants of known genotype growing in the experimental garden, flower size and shape showed the same pattern as flowers collected from unknown genotypes in natural populations. Staminate-phase flowers had significantly smaller petals, but larger corolla diameters than pistillate-phase flowers (Table 2, Fig. 4A, B). Furthermore, this pattern was the same whether or not the plants were growing in shade or sun plots; none of the flowers size measurements showed a significant treatment effect or a significant gender by treatment interaction (Table 2).

**Table 2:** Two-way ANOVA results describing the effects of treatment (shade vs. sun) and floral stage (staminate vs. pistillate) on floral traits of protandrous *S. officinalis*.

Flower Trait	Effect	df	F	P
Petal Width (mm)	Gender	1	14.04	<0.001
	Treatment	1	0.884	0.350
	Gender x Treatment	1	1.25	0.267
Petal Length (mm)	Gender	1	4.48	0.037
	Treatment	1	0.225	0.626
	Gender x Treatment	1	1.28	0.261
Corolla Diameter (mm)	Gender	1	29.04	<0.001
	Treatment	1	1.31	0.255
	Gender x Treatment	1	3.12	0.081
ABS_λ527nm_/g/mL	Gender	1	54.92	<0.001
	Treatment	1	7.60	0.007
	Gender x Treatment	1	7.26	0.008
Pinkness	Gender	1	52.85	<0.001
	Treatment	1	3.60	0.062
	Gender x Treatment	1	2.94	0.091

Flower color was significantly different in staminate- and pistillate-phase flowers as measured by both anthocyanin concentration and pinkness index (Table 2, Fig. 4C, D). Color was also affected by sun exposure, unlike the morphological traits. Flowers growing in the sun had significantly higher anthocyanin concentrations than those in the shade and had marginally significantly higher pinkness indices (Table 2, Fig. 4C, D). Pistillate-phase flowers are always pinker than staminate-phase flowers. There was a significant gender by treatment interaction for anthocyanin concentration, indicating pistillate-phase flowers turned significantly pinker in the sun than in the shade, with pinkness index showing the same pattern, although it was not statistically significant (Table 2, Fig. 4C, D).

#### UV Nectar Guides

**Figure 5:**
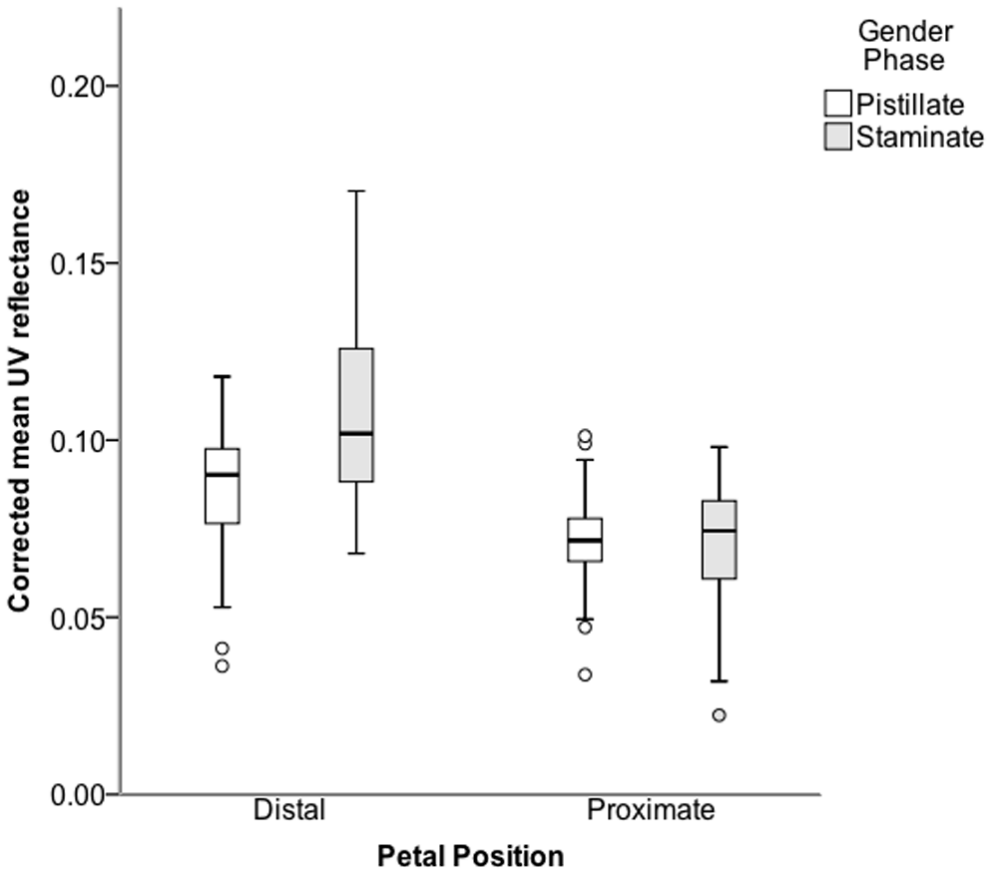
Possible UV nectar guides in *S. officinalis*. Differences in the UV reflectance at proximal and distal ends of petals of *S. officinalis* in staminate- and pistillate-stage flowers. Boxes represent the median and quartile ranges for each variable. Open circles represent values that lie between 1.5 and 3 box lengths from the end of the box. The average percentage of light reflectance in the UV range was corrected for overall amount of light reflected over the entire spectrum (see Methods).

Photographs taken in a darkroom with a UV-black light source and without the UV filter did not reveal any nectar guides for any of the sex-phases visible to the human eye. However, when the raw UV reflectance was corrected for the height of the peaks in the visible spectrum, there was a small but significant difference in the UV reflectance values of distal and proximal positions on the petal (F = 35.83, P<0.001). In addition, there was also a significant effect of gender (F = 6.60, P = 0.012) and a significant interaction between position and gender (F = 7.38, P = 0.008). In other words, staminate flowers had a higher UV ratio than pistillate flowers overall, and there was more of a contrast in reflectance between the distal and proximal petal positions in the staminate phase than there was in the pistillate phase (Fig. 5).

### Nectar production and floral age

**Figure 6:**
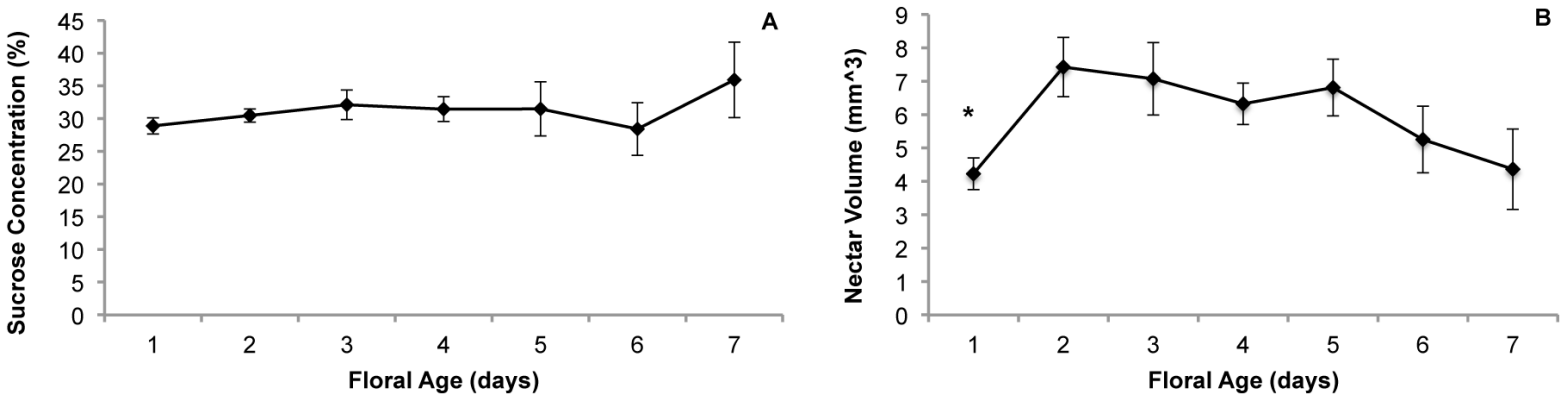
Nectar in *S. officinalis* flowers over time. Sugar concentration (A) and nectar volume (B) of flowers *S. officinalis* in a growth chamber. Flowers are staminate for days 1–2, and transition to pistillate for days 3–5, and then begin to senesce over days 6–7. Error bars represent standard error of the mean for flowers collected from 10 different plants. An asterisk (*) indicates a comparison that is statistically different from the subsequent day (α = 0.05).

Sucrose concentration in nectar collected from flowers on plants growing in the growth chamber did not change across the seven days flowers were monitored (Repeated measures ANOVA, Greenhouse-Geisser correction, F = 0.937, df = 2.531, P = 0.474, Fig. 6A). Nectar volume was significantly lower on the first day after anthesis, and then rose on the second day when flowers are still in their staminate phase (F = 11.227, df = 1, P = 0.010). Nectar volume remained steady on days 2–5 as flowers transitioned from staminate to pistillate phase, and then started to decline slightly on days 6 and 7 and flowers began to senesce, but the difference was not statistically significant (P>0.1 for all comparisons after day 2, see Fig. 6B).

### Pollinator Observations

**Figure 7:**
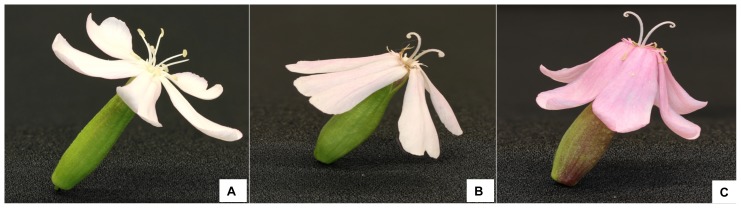
Artificial decoupling of shape and color in *S. officinalis*. Inflorescence arrays were constructed of either pale staminate flowers (A), pale pistillate flowers from plants that had been shaded (B), or pink pistillate flowers from plants growing in the sun (C).

Flowers used in the arrays differed in both color and shape. By shading plants we were able to construct arrays of inflorescences composed of pale staminate flowers, pale pistillate flowers, and pink pistillate flowers (Fig. 7).

**Figure 8:**
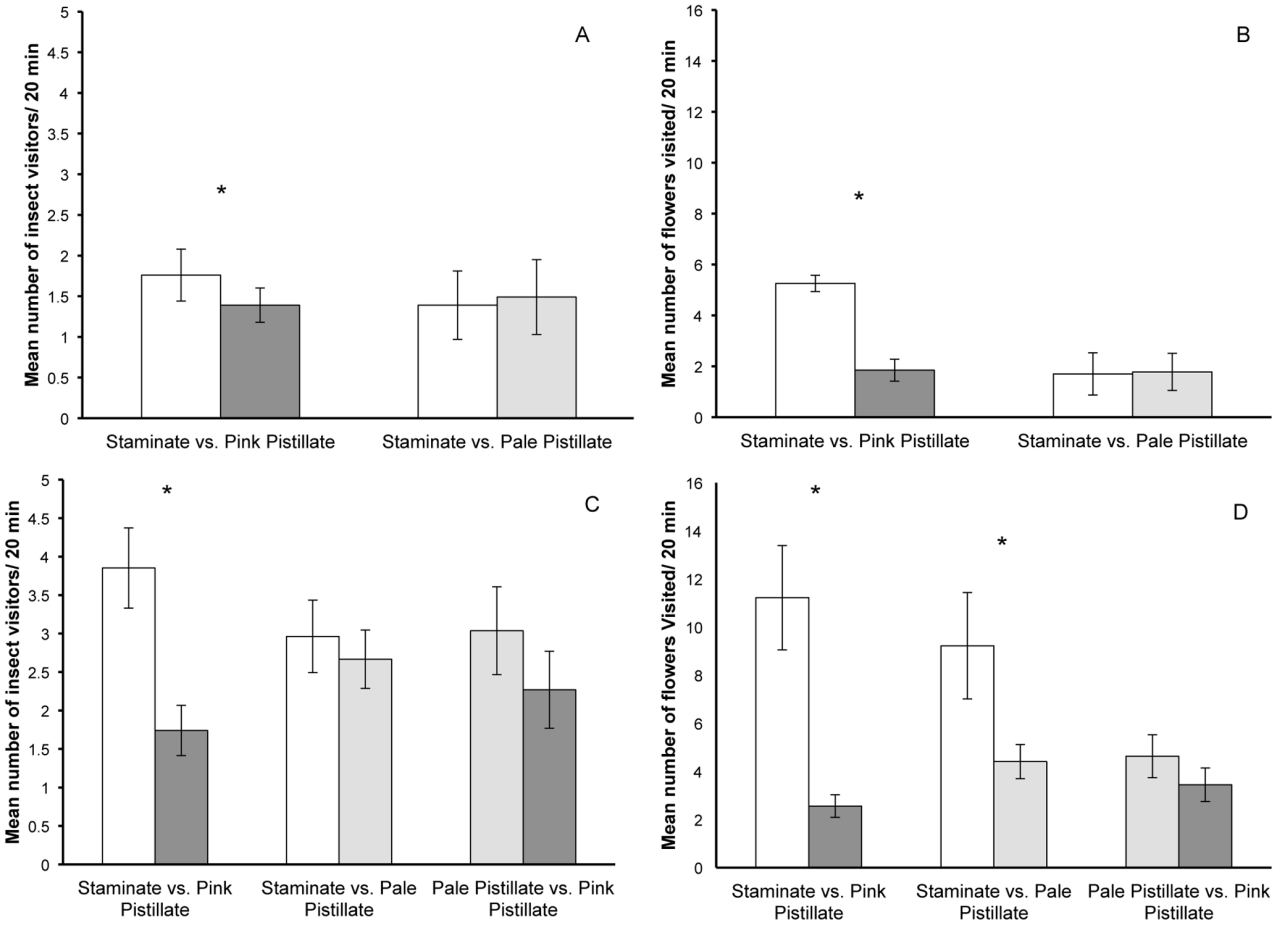
Insect preference for staminate-phase or pistillate-phase flowers of *S. officinalis*. The number of insect visitors (A) and the number of flowers visited (B) in artificial arrays of inflorescences of staminate-phase vs. pink pistillate-phase flowers and arrays of staminate-phase vs. pale pistillate-phase flowers in 2012. In 2013, the number of insect visitors (C) and the number of flowers visited (D) was recorded for 3 types of arrays: staminate-phase vs. pink pistillate-phase flowers, staminate-phase vs. pale pistillate-phase flowers, and pale pistillate-phase vs. pink pistillate-phase flowers. An asterisk (*) indicates a comparison that is statistically different (α = 0.05).

**Figure 9:**
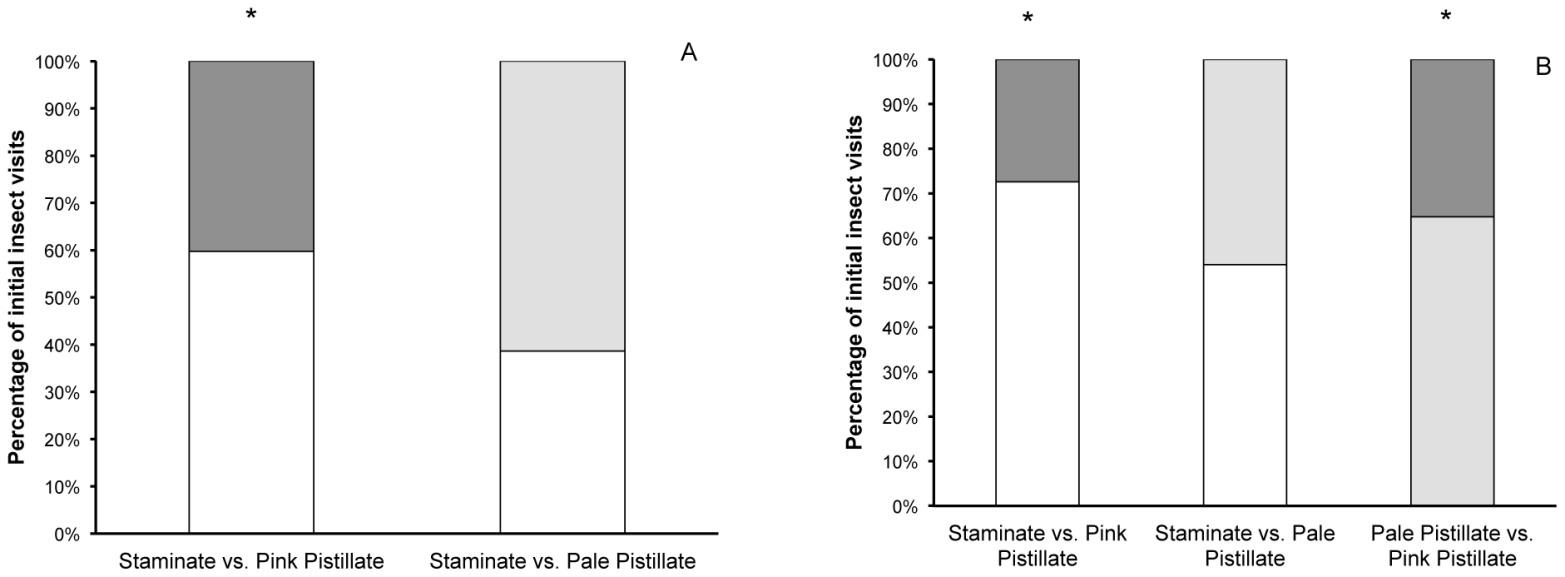
Likelihood of initial visits. The percentage of insects that initially visited artificial inflorescences of staminate-, pink pistillate-, or pale pistillate-phase flowers in pollinator observation trials in 2012 (A) and 2013 (B). Each trial type was tested against a hypothesis of each flower type receiving 50% of the first visits of an insect visitor. An asterisk (*) indicates a ratio significantly different from 50∶50 (α = 0.05).

In 2012, for trials comparing arrays of white staminate-phase and pink pistillate-phase flowers, a total of 85 pollinators were observed over 680 minutes, including honeybees, sweat bees, wasps, bumblebees, and butterflies. In all comparisons, pollinators preferred staminate-phase flowers to pink pistillate-phase flowers (Fig. 8A, B and Fig. 9A). Staminate arrays received more insect visits per 20 min interval (t = 2.23, P = 0.033), had more flowers visited (t = 2.80, P = 0.009) and insects were more likely to visit a staminate array first (P = 0.012). In contrast, when arrays of white staminate-phase were compared to pale pistillate-phase flowers, pollinator activity was similar on the two kinds arrays. None of the comparisons were significantly different (Fig. 8A, B and Fig. 9A).

In 2013, pollinators again preferred staminate-phase flowers to pink pistillate-phase flowers (Fig. 8C, D and Fig. 9B). Staminate arrays received more insect visits per 20 min. period (t = 4.01, P<0.001), had more flowers visited (t = 4.76, P<0.001) and insects were more likely to visit a staminate array (P<0.001) first. When arrays of staminate-phase flowers were compared with arrays of pale pistillate-phase flowers, staminate arrays did not have a greater number of insect visits (t = 0.788, P = 0.438) nor were they more likely to receive an initial visit from an insect (P = 0.305), but the number of flowers visited was significantly higher (t = 2.54, P = 0.018) (Fig. 8C, D and Fig. 9B). Finally, when pale pistillate arrays were presented with pink pistillate arrays, the pale arrays were more likely to receive an initial visit (P = 0.001, Fig. 9B), but did not receive more overall insects visits (t = 1.70, P = 0.101), and had marginally more flowers visits, although this difference was not statistically significant (t = 1.85, P = 0.077) (Fig. 8C, D and Fig. 9B).

The total number of insects visiting the staminate vs. pink pistillate and the staminate vs. white pistillate trials was lower in 2012 than in 2013 (F = 18.77, P<0.001), likely due to the 2012 drought in the Midwest, but there was not an effect of trial type on the number of insect visitors (F = 0.038, P = 0.847) or interaction between year and trial type (F = 0.714, P = 0.400). Therefore, within each year, both trial types received the same number of total insect visitors and the differences seen between inflorescence arrays within trials is not due to a difference in overall insect visitors. Likewise, in 2013, there was no overall effect of trial type on the total number of insects visiting each of the three trial types that were performed in that year (F = 0.072, P = 0.931).

### Seed Set

#### Experimental Garden 2012

**Figure 10:**
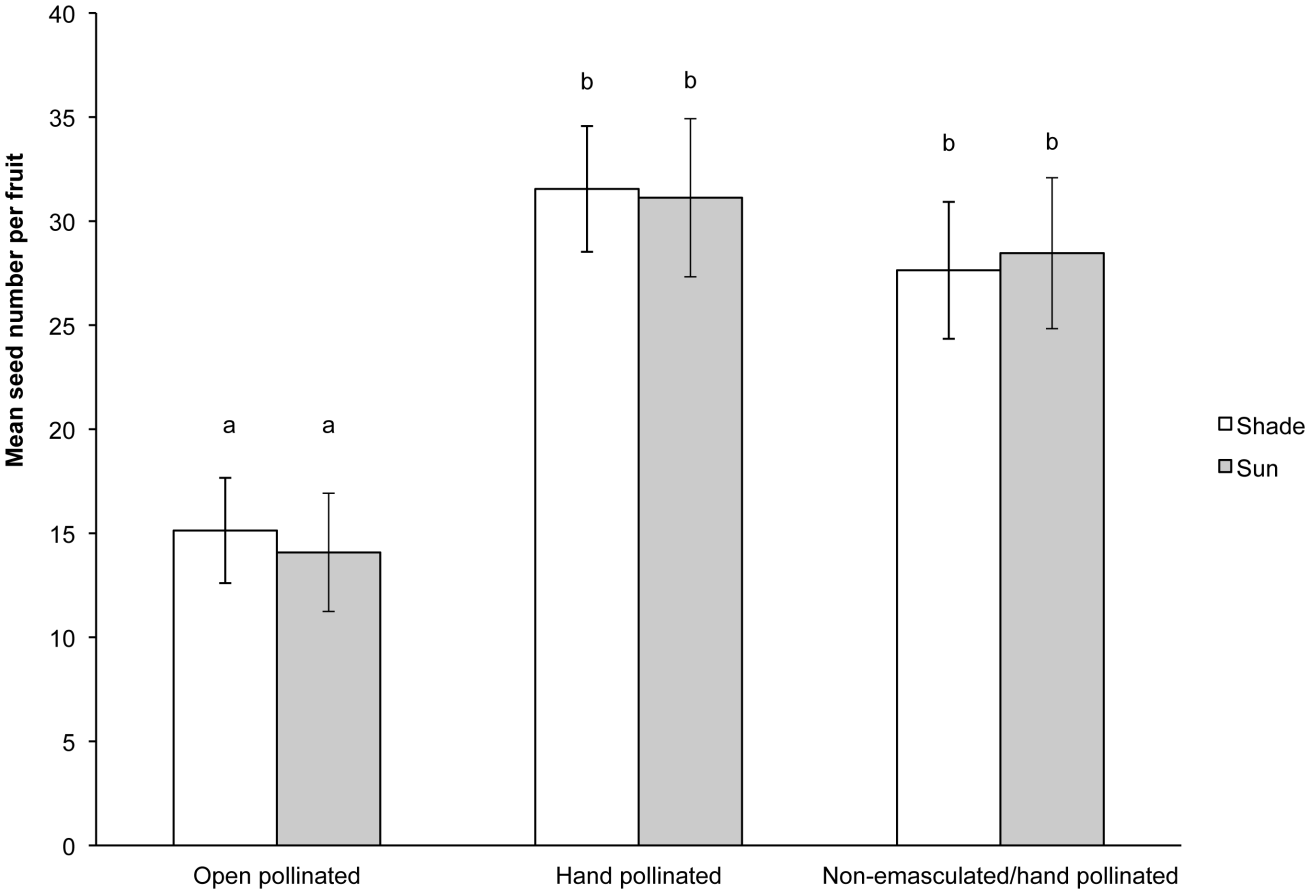
Pollen limitation. Seeds produced by emasculated open-pollinated and hand-pollinated flowers and intact hand-pollinated flowers of *S. officinalis* growing in shaded and sun exposed plots in 2012. Different letters above the bars indicate statistically significant differences (α = 0.05).

In 2012, plants growing in the experimental garden experienced severe pollen limitation. Flowers that were emasculated and left to be open pollinated produced significantly fewer seeds than flowers that were emasculated and hand pollinated in both the shade and sun plots (t = −4.85, P<0.001 and t = −4.25, P<0.001, respectively, Fig. 10). Furthermore, emasculation had no effect on seed set; emasculated and intact hand-pollinated flowers produced similar numbers of seeds per fruit regardless of treatment (t = 0.964, P = 0.346 and t = 0.512, P = 0.613, Fig. 10).

**Figure 11:**
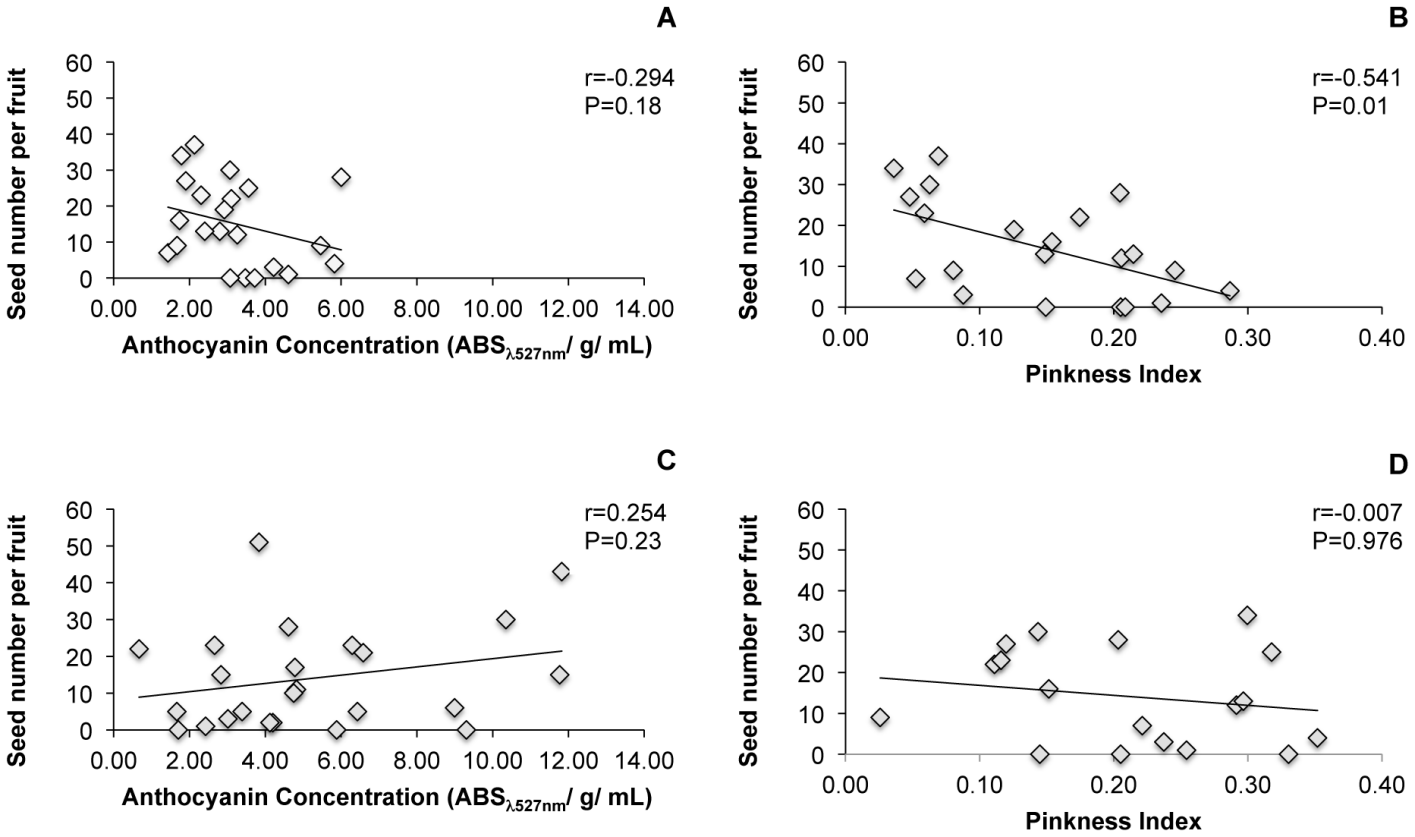
Correlation between flower color and seed production. The relationship between the number of seeds produced by emasculated open pollinated flowers and the color of a pistillate phase flower collected from the same plant in shaded plots (A and B) and sun-exposed plots (C and D). Best-fit lines added for clarity.

The number of open-pollinated seeds produced in emasculated flowers was not correlated with any of the measures of flower size in pistillate-phase flowers (P>0.05 for all pairwise tests). There was, however, a significant negative correlation between pinkness index and seed production in shaded plants (Fig. 11B). The correlation between anthocyanin concentration and seed production was also negative for these plants, but this difference was not statistically significant (Fig. 11A). In the sun-exposed plants, seed production was not significantly correlated with either anthocyanin concentration or pinkness index (Fig. 11C, D).

#### Experimental Garden 2013

Despite cooler weather, greater rainfall, and greater insect activity on our experimental arrays, plants in the 2013 garden again showed pollen limitation of seed set. Emasculated open-pollinated flowers produced significantly fewer seeds than emasculated hand-pollinated flowers in both the shade (22.91±1.47 vs. 33.11±1.61, P<0.001) and the sun (26.27±1.65 vs. 33.85±1.78, P<0.001).

Sun and shade treatments affected the anthocyanin concentration of petals in the same manner as before, with flowers from the sun treatment having significantly higher anthocyanin concentration than those from the shade treatment (P<0.001, Table 3). Furthermore, the four genotypes in the pink group had significantly higher anthocyanin concentration in their petals than those four genotypes in the pale group (P<0.001), however, there was some variation among the genotypes within the pale and pink groups (P = 0.001, Table 3). Finally, both pale and pink genotypes showed a similar response to sun exposure as there was not a significant interaction between group and treatment (P = 0.141).

**Table 3:** Nested ANOVA results examining the effects of treatment (shade vs. sun) and genotype (nested within pale vs pink sets) on anthocyanin concentration and seed set in open-pollinated and hand-pollinated flowers of *S. officinalis*.

	ABS_λ527nm_/g/mL	Open-pollinated seed set	Hand-pollinated seed set
**Treatment (sun vs shade)**	45.62 (1)	3.23 (1)	(1)
	**P<0.001**	P = 0.072	P = 0.978
**Set (pale vs pink)**	111.04 (1)	7.13 (1)	8.92 (1)
	**P<0.001**	**P = 0.008**	**P = 0.003**
**Genotype (nested within set)**	23.43 (6)	22.56 (6)	78.82 (6)
	**P = 0.001**	**P = 0.001**	**P<0.001**
Treatment*Set	2.17 (1)	0.15 (1)	3.87 (1)
	P = 0.141	P = 0.700	**P = 0.049**
Treatment*Genotype	12.26 (6)	2.69 (6)	5.55 (6)
	P = 0.057	P = 0.847	P = 0.476

Values in the table are the Wald Chi-square statistic (df) and the probability value for each effect in the test for model effects.

**Figure 12:**
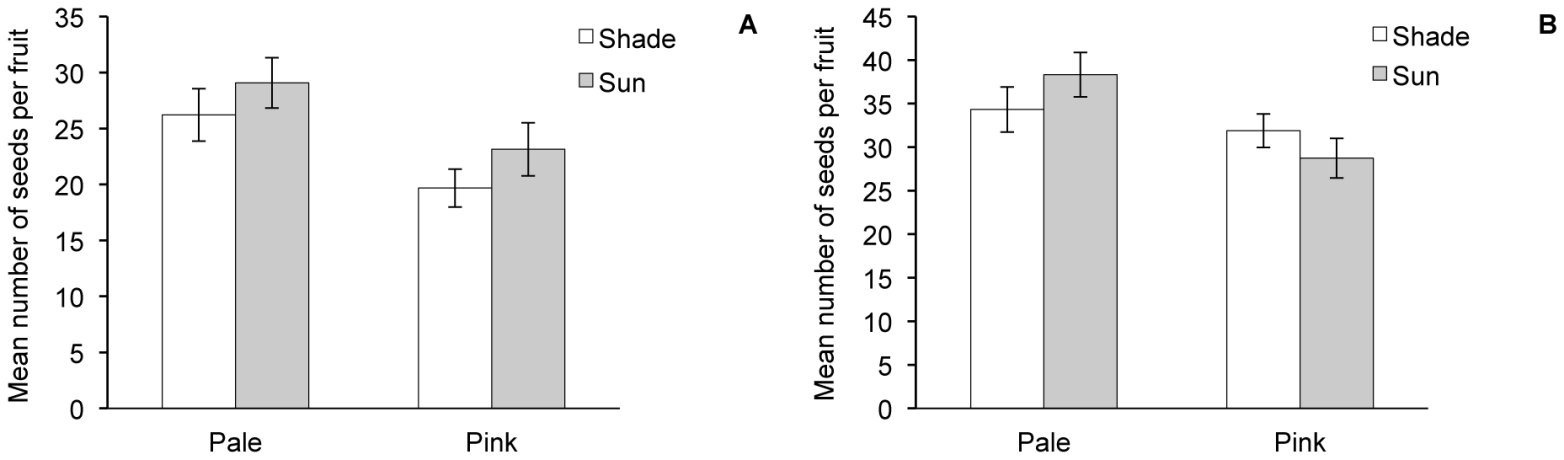
Seed production in pale vs. pink genotypes. The number of seeds produced in open-pollinated (A) and hand pollinated (B) flowers by genotypes producing whiter flowers (“Pale”) and genotypes producing pinker flowers (“Pink”) under shade and sun treatments.

In addition to having less-pink flowers, the pale genotypes produced significantly more seeds per fruit in both the open-pollinated and hand-pollinated flowers (Fig 12A and 12B, P = 0.008 and P = 0.003, Table 3). There was a trend, but non-significant effect of treatment on seed set in the open-pollinated flowers, with those flowers in the sun producing more seeds than those in the shade (Fig. 12A, P = 0.072, Table 3), perhaps due to higher pollinator activity in the sun plots. This effect disappears in the hand-pollinated flowers (Fig. 12B, P = 0.978), and there is a slight interaction effect between group and treatment (Fig. 12B, P = 0.049, Table 3), indicating that seed set in the pale and pink genotypes are being affected differently by the sun and shade treatments.

## Discussion

### Dichogamy and Floral Morphology

In *Saponaria officinalis*, dichogamy does not simply entail a shift from a staminate to a pistillate stage within the protandrous flowers. Individual flowers undergo a transformation in size, shape, and color as well, therefore showing sexual dimorphism between the gender phases. Sexual dimorphism is commonly defined as differences between males and females in secondary sexual characteristics [13], [36]. This study confirms that selection can also act to influence the evolution of the two discrete reproductive strategies when the genders are combined in hermaphroditic flowers [12].

Delph and Herlihy [14] showed that sexual selection or sex-specific selection for fecundity or survival can lead to sexual dimorphism. Floral traits that increase pollinator attraction should evolve under selection for increased male fitness more so than for selection for increased female fitness. However, selection can also act on pollinator attraction through female function if pollen is limiting [18]–[22]. In *S. officinalis*, staminate flowers have wider corollas than pistillate flowers (Fig. 2). This change in display size is not the result of senescence; in fact, the petals become longer, wider, and heavier as the flower transitions to pistillate phase (Table 1). Rather, the decreased display size of the pistillate flowers is due to a change in shape, as the petals reflex toward the corolla tube. If interference between male and female functions was important in the evolution of protandry in this species, additional changes in floral morphology would also be expected to occur that would further reduce interference between stamens and pistils [1]. The reflexing of the petals in the pistillate stage also pulls the stamens further from the styles, in agreement with this prediction (Fig. 2).

As in a previous study [31], pistillate flowers had higher anthocyanin concentrations than staminate flowers, and this difference increased when plants were exposed to the sun (Fig. 4C). In addition to measuring anthocyanin concentration by the absorbance values of petal extracts, we also measured flower color by capturing the reflectance spectra of intact petals. Since the petals remain intact, reflectance spectroscopy allows one to study the color of petals more as they are actually seen by pollinators [37]. Just as anthocyanin concentration increased with sun exposure, so did the pinkness index of the reflectance spectra (Fig. 4D). The positive correlation between anthocyanin concentration and pinkness index (Fig. 3) indicates that it is likely the increase in anthocyanins that leads to the observed change in flower color. The increased anthocyanin production under the sun treatment was independent of changes in floral morphology related to dichogamy. There was no difference between any of the size measurements of flowers in the sun and shade treatments (Fig. 4A, B).

No nectar guides were detectable to the naked eye under black light in *S. officinalis*. However, when comparing proximal and distal ends of the petals, the reflectance spectrometer detected higher UV reflectance in staminate-phase flowers than pistillate-phase flowers. Furthermore, there was a larger discrepancy in UV reflectance between the distal and proximal positions in the staminate phase than there was in the pistillate phase (Fig. 5). The percent reflectance values are low (<10%) for *S. officinalis* (Fig. 5). But if small bees and flies, the main daytime pollinators of *S. officinalis*, can detect this difference, the differences between pistillate-phase and staminate-phase flowers may play an influential role in shaping pollinator discrimination between the gender phases. The greater difference between the petal positions in staminate-phase flowers suggests that the nectar guides are more prominent (there is more difference between the light and dark areas) when the flowers are in the staminate phase. The dark center may also be used as a contrast against the pollen grains, which are known to reflect ultraviolet light in many flowers [38]. Regardless, changes in floral color in the visible spectrum do not have to be associated with changes in the UV spectrum. In a review of species displaying floral color change, Weiss [28], found no evidence of changes occurring in the UV spectrum.

Carlson and Harms [25] reviewed over 20 studies of dichogamous species that reported greater nectar production during the staminate phase, and Varga et al. [27] reported greater nectar production in staminate phase hermaphrodites of the gynodioecious *Geranium sylvaticum*. Our study of *Saponaria officinalis* is unusual in that we did not detect differences in sugar concentration or nectar volume (after day 1) that corresponded with floral age or gender phase (Fig. 6). Our study was the first to consider changes in daily nectar quantity over the floral lifetime in *S. officinalis*. A previous study of nectar dynamics in this species [39] concluded that covered and uncovered flowers showed a daily rhythm of nectar production, with an increase in nectar production at night and early morning hours. This cyclical variation may correspond to the activity of nocturnal pollinators (moths), which are more likely to be nectar gatherers than diurnal small bees and flies [40].

### Pollinator Discrimination

Diurnal pollinators preferred staminate flowers over pink pistillate flowers in both 2012 and 2013 in terms of the total number of insects visiting, number of flowers visited, and number of initial visits (Fig. 8 and 9). However, when the contrast in color between the arrays was reduced by comparing staminate arrays to pale-pistillate arrays from shaded plants, pollinators no longer discriminated against pistillate flowers, in general (Fig. 8 and 9). In 2013, we compared pale pistillate flowers arrays to pink pistillate flower arrays. Again, there was a trend for pinker flowers to receive fewer insect visits and have fewer flowers visited, but these differences were not statistically significant (Fig. 8). However, the pinker arrays received a significantly lower percentage of initial insect visits than the pale pistillate flowers (Fig. 9B). Taken together, our pollinator observations indicate that pollinators use color to discriminate among flowers more than differences gender phase, size or shape.

Our results are only applicable to the diurnal pollinators that were observed in this study. *S. officinalis* is also pollinated by nocturnal moths, but in a study by Jabbari et al. [31], when either set of pollinators were excluded from inflorescences, there was no difference in fruit or seed set compared to control flowers. Therefore, diurnal pollinators contribute substantially to seed set in this species. The diurnal bees and flies are more likely to be gathering pollen, whereas the nocturnal moths are more likely to gather nectar [40]. Bertin and Newman [1] found that simultaneous hermaphroditism was more likely to associated with pollen-reward species and dichogamy associated with nectar-reward species. They hypothesized that these associations might be related to the difficulty of attracting pollen-collecting pollinators to stamen-less pistillate phase flowers. Therefore, selection may reduce dimorphism between gender phases to ensure pollination of pistillate-phase flowers through mimicry in species that only use pollen as a reward [23]. We did find substantial pollen limitation of seed set in *S. officinalis*, indicating pollinator activity is reducing female fitness. However, since we did not find a difference in nectar production between the gender phases, (Fig. 6), we predict that we would detect weaker preferences among the nocturnal than among diurnal pollinators in *S. officinalis*.

### Seed Set and Female Fitness

Low pollinator activity was found to limit seed set in our experimental gardens in 2012 and 2013 (Fig. 10). Therefore, pollinator discrimination against pistillate-phase flowers has the potential to reduce female fitness. Under pollen limitation, selection should lead to a correlation between attractive structures and female fitness [19]. In 2012, we found that there was a negative correlation between the pinkness of pistillate-phase flowers that a plant produced and the number of seeds that were produced in emasculated, open-pollinated flowers (Fig. 11) in the shaded plants, though not in the sun-exposed plants. Thus, diurnal pollinator preferences for pale flowers may have led to predictable differences female fitness in plants with reduced sun exposure.

In 2013, the garden contained two subsets of genotypes selected from the 2012 plants: one set that produced paler pistillate flowers in 2012 and one set that produced pinker pistillate flowers in 2012. As they did in 2012, pinker genotypes produced higher levels of anthocyanins in 2013 than pale genotypes (Table 3), indicating some heritability for this trait. As predicted from the pollinator observations, the pinker genotypes also produced fewer seeds than the paler genotypes in open-pollinated flowers in both the shade and sun treatments (Fig. 12A, Table 3).

The difference in seed production between the pale genotypes and the pink genotypes is not solely a function of pollinator visitation. The pink genotypes also produced fewer seeds in hand-pollinated flowers (Fig. 12B) in 2013, indicating that variation in seed production is due in part to some other difference between the two sets of genotypes, such as pleiotropic effects of genes involved in anthocyanin production, or increased investment in pigment production reducing investment in seed production [41]. The reduction in seed set in the pink genotypes was not as drastic in the hand-pollinated flowers as it was in the open-pollinated flowers (Fig. 12). This indicates that pollinator preferences for pale flowers did influence the difference in seed set between the pink and pale sets of genotypes in 2013. In the hand-pollinated flowers, there is a significant interaction between the pale/pink sets and treatment (Table 3). In this case, the pink genotypes produced fewer seeds when in the sun than when they were in the shade (Fig. 12B).

## Conclusions

Staminate and pistillate phase flowers of *S. officinalis* are dimorphic in size, shape, and color. In addition, pollinators discriminate against pistillate-phase flowers based on their color. This pollinator discrimination may lead to a reduction in seed set in those individuals that produce pinker pistillate flowers and, hence, have increased dimorphism in flower color. If the morphological changes in the protandrous flowers of *S. officinalis* actually reduce female fitness, the question remains: why is this dimorphism maintained?

The evolution of sexual dimorphism in plants such as *S. officinalis* involves complex interactions between differing selection pressures for male and female reproductive roles, indirect selection of correlated traits, selection for reproductive assurance, and is mediated by the degree to which traits are under the control of shared genetic architecture [9], [13]–[14].

Dichogamy and gender dimorphism between the staminate and pistillate phases may have multifaceted effects on plant fitness beyond pollinator attraction and seed set. Dichogamy will reduce within-flower self-fertilization, but if multiple flowers are open on a plant, self-fertilization through geitonogamy is still likely [4], [42]. A pollinator preference for either gender might reduce the level of geitonogamy by reducing the number of flowers visited by a single pollinator. Color change in *S. officinalis* may be acting similarly to other species that retain color-changed flowers after they are sexually viable, by increasing display size to attract pollinators from a distance but then directing pollinators to specific flowers when they approach [28]. This scenario would be more likely if pistillate flowers were more likely to be visited first, which does not occur (Fig. 9), or if plant architecture was structured so that pollinators tended to move from female flowers to male [43], but we would need more information about how dichogamy could reduce geitonogamy in loosely arranged inflorescences like those found in *S. officinalis* and the interaction between gender preference of pollinators and potential preference for larger floral displays.

Another unanswered question in this system is the effect of the floral morphology changes on male fitness. It is presumed that increased pollinator visits would lead to increased seed siring. So in this respect, increased attractiveness of staminate flowers matches the predictions of Bateman's Principle [17]. In addition, increased display size leading to increased pollinator attraction is predicted to enhance male fitness more than female fitness. For example, in monoecious *Sagittaria trifolia*, Huang et al. [44] found that pollinators preferred male flowers to female flowers on inflorescences of the same size. They also found that pollen removal (male fitness) was limited by pollinator visitation, but pollen receipt (female fitness) was not. If anthocyanin production in pistillate flowers of *S. officinalis* is constrained by pleiotropy, perhaps delaying pigment production until flowers are past the staminate stage serves to increase pollinator visitation to reproductively male flowers. The biosynthetic pathway of anthocyanin production has been well characterized and various steps in the pathway have been identified as points that regulate the production of pigment in response to ontogenetic and environmental changes in several species [45]–[49]. We are currently examining the gene expression of enzymes involved in early and late stages of anthocyanin production across the floral stages, environmental conditions (sun versus shade), and across genotypes to determine the molecular basis of floral color change in *S. officinalis*.

The increase in pinkness of pistillate flowers may simply be a non-adaptive by-product. Anthocyanin pigments have been found to protect against photoinhibition in vegetative tissues (e.g., [50]), by serving as a sunscreen to block blue-green and UV light, producing varying colors (red, purple, or blue) based on pH levels, and helping to fight heat stress and desiccation [41], [51]. Pigmentation in flowers may be a result of a correlation with pigment production in vegetative tissue, as described in Armbruster [52]. If genetic variation exists in the degree of color change, it has the potential to be shaped by selection. In this study we presented evidence that some genotypes produce pinker flowers than others. This is part of a larger study being conducted to determine genotypic differences in overall flower color, flower color response to environmental conditions, and several vegetative traits. Studies of sexual dimorphism are complicated by the fact that it can be difficult to disentangle the effects of sex-specific selection, sexual selection, and correlated genetic responses to selection [12]–[14], [36]. *Saponaria officinalis* provides an opportunity to study the fitness effects of dimorphism of reproductive traits while reducing the confounding effects of genetic differences between individuals of different genders, since both reproductive strategies are expressed in the same individual.
