# Correction: Sexual Dimorphism of Staminate- and Pistillate-Phase Flowers of *Saponaria officinalis* (Bouncing Bet) Affects Pollinator Behavior and Seed Set

**DOI:** 10.1371/journal.pone.0102865

**Published:** 2014-07-21

**Authors:** The PLOS ONE Staff

The formula for “Pinkness Index” in the “Correlation between Reflectance and Anthocyanin Concentration” section of the Materials and Methods is incorrectly displayed. The correct formula, provided by the Authors, is shown below.

**Figure:**
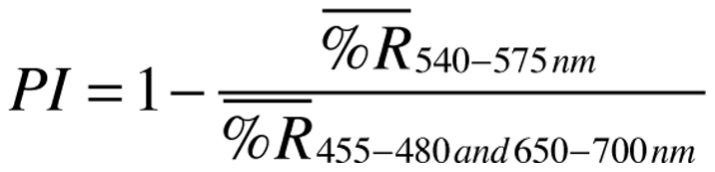


## References

[1] Davis SL, Dudle DA, Nawrocki JR, Freestone LM, Konieczny P, et al (2014) Sexual Dimorphism of Staminate- and Pistillate-Phase Flowers of *Saponaria officinalis* (Bouncing Bet) Affects Pollinator Behavior and Seed Set. PLoS ONE 9(4): e93615 doi:10.1371/journal.pone.0093615 24690875 10.1371/journal.pone.0093615 PMC3972141

